# Opportunistic infections in immunodeficient populations.

**DOI:** 10.3201/eid0403.980321

**Published:** 1998

**Authors:** J E Kaplan, G Roselle, K Sepkowitz

**Affiliations:** Centers for Disease Control and Prevention, Atlanta, Georgia, USA.


### Opportunistic Infections in Immunodeficient Populations

**Jonathan E. Kaplan,* Gary Roselle,† and Kent Sepkowitz‡**
*Centers for Disease Control and Prevention, Atlanta, Georgia, USA;
†Veterans Administration Medical Center, Cincinnati, Ohio, USA; and
‡Memorial Sloan-Kettering Cancer Center, New York, New York, USA

Opportunistic infections occur with greater frequency or severity in patients with impaired host defenses. Growing numbers of HIV-infected persons, transplant recipients, and elderly persons are at increased risk.

Alison Grant, London School of Hygiene and Tropical Medicine, discussed opportunistic infections due to HIV. In 1997, more than 30 million HIV-infected persons lived in the world, with more than two thirds of them in sub-Saharan Africa and an additional 20% in Asia and Latin America. Assessments of the prevalence and incidence of opportunistic infections in these areas and comparability of the available data are hampered by limited access to care, diagnostic capabilities, and surveillance data. Despite these limitations, we know that tuberculosis (TB) is the most frequent serious opportunistic infection in the developing world. Other such infections common in sub-Saharan Africa include septicemia (of which nontyphoid salmonella is the most common cause), toxoplasmosis, and bacterial pneumonia. *Pneumocystis carinii* infection, for unknown reasons, is uncommon among adults in East and West Africa but appears to be more common in South Africa. *Penicillium marneffei* infection, common in Thailand, is an example of an opportunistic infection of importance in a specific region; risk factors in these regions are largely unknown. Additional challenges are posed by the different HIV subtypes in the developing world and the possibility that some may be associated with a differential risk for opportunistic infections. Prevention efforts in developing countries have been limited. More work is needed to evaluate prophylactic regimens appropriate to different regions. Prevention of TB with isoniazid; of pneumocystosis, toxoplasmosis, and some bacterial infections with cotrimoxazole; and of pneumococcal infections with 23-valent pneumococcal vaccine have potential.

Robert Hogg, University of British Columbia, discussed the remarkable changes in the natural history of HIV in North America, specifically in British Columbia, as a result of highly active antiretroviral therapy (HAART). Of more than 5,000 HIV-infected persons receiving care in British Columbia, more than 2,000 are receiving HAART. HIV viral loads have been reduced to undetectable levels in approximately half of these patients, with corresponding decreases in the incidence of opportunistic infections, hospitalizations, and deaths. However, even for persons who have access to the therapy, these successes may be short-lived as resistance to HAART becomes more widespread. HAART use has resulted in new syndromes that may occur soon after therapy, probably representing preexisting, subclinical infections that are unmasked by the immunologic improvement that accompanies HAART; these syndromes include lymphadenitis associated with *Mycobacterium avium* complex, cytomegalovirus retinitis, and miliary TB on chest X-ray. Hepatitis C infection, common in HIV-infected injection drug users, may pose increasing problems as coinfected persons live longer. Therefore, surveillance for old and new syndromes remains critical even with the reduced incidence of opportunistic infections that has been associated with HAART.

Robert Rubin, Harvard University and Massachusetts Institute of Technology, discussed opportunistic infections in hematopoietic stem cell (bone marrow) and solid-organ transplant recipients; the number of these transplant recipients has increased dramatically in the United States in the past decade. The opportunistic infections in these patients originate from endogenous flora (e.g, invasive candidiasis), from the general (nonhospital) environment (e.g, histoplasmosis, TB, disseminated strongyloidiasis), or from the hospital environment (e.g, aspergillosis, legionellosis, and infections with vancomycin-resistant enterococci or multiply resistant gram-negative bacteria). These infections characteristically occur in a time-dependent pattern posttransplant, corresponding with the nature of the immunodeficiency. For example, in bone marrow transplant recipients, infections within 1 month of transplantation (pre-engraftment) occur as a result of neutropenia and disruption of mucosal surfaces; infections that occur in the second or third months are due to deficiencies in cell-mediated immunity and are more frequent in the setting of graft versus host disease. In solid-organ transplant recipients, infections within the first month are generally associated with technical problems related to surgery; infections that occur later are due to immunodeficiency associated with immunosuppressive therapy. These timetables are useful in that infections that are unusual or occur outside the expected time frame may serve as sentinels for emerging opportunistic infections. Research priorities in this area include development of therapies that will enhance successful transplantation without increasing the risk for opportunistic infections, strategies to reduce the risk of drug-resistant opportunistic infections, and greater understanding of the role of cytokines in the relationship between graft versus host disease and opportunistic infections.

Carol Kauffman, University of Michigan and the Ann Arbor Veterans Administration Medical Center, discussed infections in the elderly, a population that is increasing in the United States and worldwide. Persons ≥65 years of age already constitute approximately one eighth of the U.S. population; this proportion is expected to double in the next 50 years. Elderly persons have defects in T-cell immunity that result in increased incidence and death from TB. B-cell defects result in increased susceptibility to *Streptococcus pneumoniae* and respiratory syncytial virus and a decreased response to 23-valent pneumococcal vaccine. Elderly persons are at increased risk for cancer, so various treatments associated with immunosuppression (such as organ transplantation and aggressive cancer chemotherapy) are increasingly being used in this population. Chronic corticosteroid therapy is frequently used for treatment of temporal arteritis. Although HIV infection is relatively uncommon in the elderly, when it does occur, it is likely to go undiagnosed. Because of higher rates of hospitalization, elderly persons are more susceptible to nosocomial infections (including those caused by antibiotic-resistant organisms). Moreover, the elderly are more likely to reside in long-term care facilities, which may serve as sources or amplifiers of infections such as influenza. Susceptibility to infection may be further increased by malnutrition, diabetes, and chronic renal failure. Finally, healthy, more affluent older persons are at risk for infections associated with travel.

In summary, opportunistic infections are a threat in the increasing populations of immunocompromised persons. In these populations, opportunistic infections pose challenges for surveillance and determination of risk factors, including those for infection with antibiotic-resistant organisms.

